# Comparison of the diagnostic accuracy of cone beam computed tomography and radiography for scaphoid fractures

**DOI:** 10.1038/s41598-018-22331-8

**Published:** 2018-03-02

**Authors:** Jakob Neubauer, Matthias Benndorf, Claudia Ehritt-Braun, Kilian Reising, Tayfun Yilmaz, Christopher Klein, Horst Zajonc, Elmar Kotter, Mathias Langer, Sebastian Moritz Goerke

**Affiliations:** 1 Department of Radiology, University Medical Center Freiburg, Hugstetter Straße 55, 79106 Freiburg, Germany; 2 Department of Orthopaedics and Trauma Surgery, University Medical Center Freiburg, Hugstetter Straße 55, 79106 Freiburg, Germany; 3 Department of Plastic and Hand Surgery, University Medical Center Freiburg, Hugstetter Straße 55, 79106 Freiburg, Germany; 4 Department of Radiology, Ortenau Klinikum Offenburg-Gengenbach, Ebertplatz 12, 77654 Offenburg, Germany

## Abstract

The aim of this study was to evaluate and compare the diagnostic accuracy, the inter-rater agreement and raters’ certainty of cone beam computed tomography (CBCT) and radiography for the detection of scaphoid fractures. Our hypothesis is that the CBCT has a higher diagnostic accuracy for scaphoid fractures than radiography. We retrospectively analysed patients who underwent both radiography and CBCT examinations within 4 days to rule out a scaphoid fracture over a 2-year period in our institution. 4 blinded radiologists and orthopaedic surgeons independently rated the images regarding the presence of a scaphoid fracture. The reference standard was evaluated by two radiologists in a consensus reading. Inter-rater correlation was evaluated, pooled sensitivity, specificity, positive and negative predictive values were calculated and compared. 102 patients met the inclusion criteria. 52% of them had a scaphoid fracture. The inter-rater correlation was higher in the CBCT compared to radiography (P < 0.001). Sensitivity, specificity, positive and negative predictive values were higher for CBCT than for radiography (P < 0.019). Observers’ fracture classifications showed a higher correlation with the reference standard in the CBCT. Observers’ certainty for fracture detection and classification were higher in the CBCT. CBCT shows a higher diagnostic accuracy for scaphoid fractures than radiography.

## Introduction

Diagnostic accuracy of plain radiography for scaphoid fractures is known to be low^1^. Especially on the initial X-ray scaphoid fractures may be occult, which may delay therapy^2^. However, if a scaphoid fracture is not adequately treated there is a risk of healing complications and possible degenerative changes^3^. Textbooks therefore recommended further clarification by means of follow-up imaging or sectional imaging in the case of negative X-ray images and the continued clinical concern for fracture^4^. Due to its high spatial resolution and potentially low radiation exposure, cone-beam computed tomography (CBCT) could be an ideal candidate the further diagnostic workup in this mostly young patient group^5^.

CBCT is increasingly applied in musculoskeletal imaging^6–8^. Although initial studies have suggested that CBCT allows imaging with lower doses than multidetector computed tomography (MDCT)^5,9^, recent studies show that both modalities allow imaging at low-dose settings^10,11^. However, in the case of musculoskeletal CBCT, it is also possible to shield the radiation-sensitive organs from radiation due to scanner design, which can further reduce the effective dose of the examinations^12^. Several studies have demonstrated CBCT’s ability to reliably examine the wrist, including arthrography or diagnosis of distal radius fractures^13^. For the diagnostic accuracy of scaphoid fractures, however, no significant difference between the sensitivity of radiographs and CBCT could be found in an initial study, which applied MRI as a reference standard^14^. This recent finding is in contrast to our clinical experience.

The purpose of our study was therefore to evaluate and compare the diagnostic accuracy of CBCT and radiography for the detection of scaphoid fractures. Our hypothesis is that the CBCT has a higher diagnostic accuracy for scaphoid fractures than radiography. We additionally investigate the inter-rater correlation of both modalities and the certainty the readers have in the diagnosis made.

## Results

**Figure 1:**
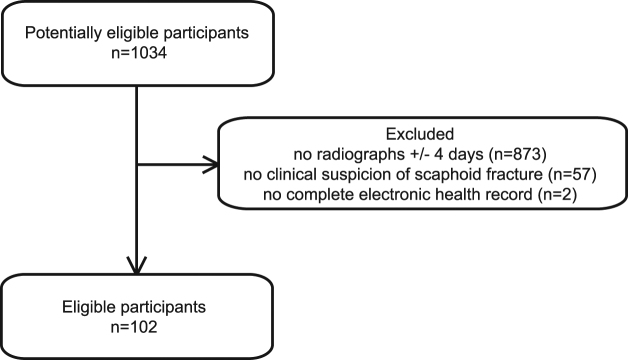
Flow of participants.

1034 potentially eligible participants had undergone CBCT imaging of the wrist in our institution from November 2012 to November 2014. 873 patients were excluded because they had no radiographs (scaphoid series) taken+/− 4 days of the CBCT examination. 57 patients were excluded because there was no clinical suspicion of scaphoid fracture. 2 patients were excluded because the electronic health record was not complete. This left 102 eligible participants (Fig. 1). We included 20 women and 82 men with a mean age of 33 years (standard deviation 16 years). According to the reference standard 53 participants (52%) had a scaphoid fracture.

The image quality ratings are shown in Table 1 and reasons for restricted image quality in Table 2. No case in the CBCT had diagnostic limitation. There was a significant difference between the image quality ratings of the two modalities (P-value < 0.001).

**Table 1:** Pooled image quality ratings.

Image quality rating	Radiography	CBCT
1 (=good)	275	79
2 (=minimal complaint without diagnostic limitation)	121	329
3 (=bad with diagnostic limitation)	12	0

**Table 2:** Reason for restricted image quality (multiple nominations were possible).

	Radiography (Number of ratings)	CBCT (Number of ratings)
Wrong rotation	101	0
Superimposing foreign material	61	0
Incomplete depiction	0	0
Physics-based artifacts	0	162
Patient-based artifacts	0	16
Scanner-based artifacts	0	297

**Figure 2:**
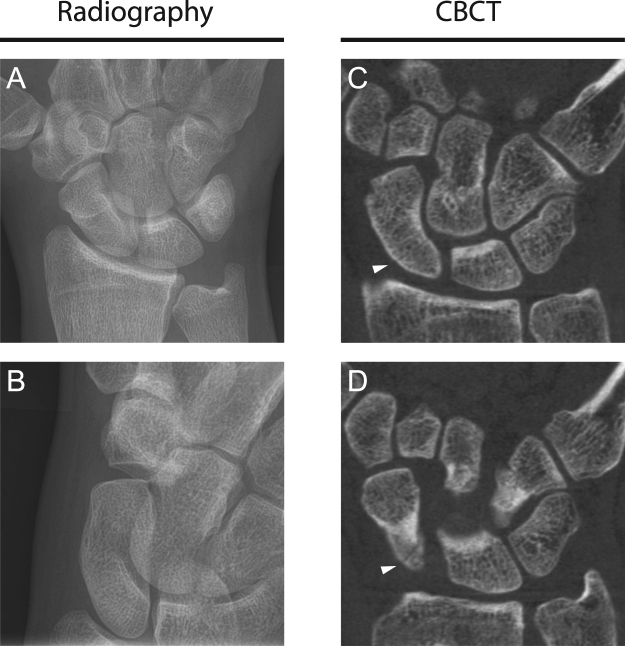
29-year-old male patient who suffered from trauma presented in the ED. Radiographs and CBCT were acquired because scaphoid fracture was suspected. No fracture is seen in the radiographs (**A** dorsopalmar, **B** dorsopalmar with ulnar deviation) whereas the coronal reconstructions of the CBCT (**C**,**D**) clearly depict the fracture (white arrowhead).

Sensitivity, specificity, positive and negative predictive values for the detection of scaphoid fractures in radiography and CBCT are shown in Table 3, imaging examples in Fig. 2. CBCT was superior to radiography in all comparisons performed (P < 0.019). Pooled contingency tables for radiography and CBCT are given in Table 4 and Table 5, respectively. Inter-rater agreement for fracture detection was 0.51 (CI 0.42–0.61) for radiography and 0.78 (CI 0.70–0.86) for CBCT (P-value < 0.001).

**Table 3:** Pooled measures of diagnostic accuracy of radiography and CBCT for scaphoid fractures.

	Radiography	CBCT	P-value
Sensitivity	0.87 (CI 0.83–0.92)	0.93 (CI 0.89–0.96)	0.019
Specificity	0.77 (CI 0.71–0.83)	0.96 (CI 0.93–0.99)	<0.001
Positive predictive value	0.80 (CI 0.75–0.86)	0.96 (CI 0.93–0.99)	<0.001
Negative predictive value	0.84 (CI 0.80–0.90)	0.92 (CI 0.89–0.96)	0.003

**Table 4:** Pooled contingency table for radiography.

	Fracture	Intact
Radiography positive	185	45
Radiography negative	27	151

**Table 5:** Pooled contingency table for CBCT.

	Fracture	Intact
CBCT positive	197	8
CBCT negative	15	188

Correlation of the fracture classification ratings with the reference standard was 0.62 (CI 0.53–0.72) for radiography and 0.78 (CI 0.72–0.85) for CBCT (P-value < 0.001). In 7% of all cases, CBCT led to upgrading in fracture classification from A- to B-fractures compared to radiography. In 8% of all cases CBCT led to downgrading in fracture classification from B- to A-fractures compared to radiography.

Median values of observers’ certainty for fracture detection were 2 in radiography and 1 in CBCT (P-value < 0.001). Median values of observers’ certainty for fracture classification were 2 in radiography and 1 in CBCT (P-value < 0.001).

## Discussion

We demonstrate that compared to radiography CBCT has a higher sensitivity, specificity, positive and negative predictive value for the detection of scaphoid fractures. Inter-rater agreement and the correlation of fracture classification with the reference standard were higher for CBCT. Observers were more confident in their fracture detection and classification using CBCT. Obviously the cross-sectional imaging technique, which allows a superimposition-free representation of the bony structures of the wrist, is better suited for the diagnosis of scaphoid fractures than the projection method radiography.

Studies with MDCT already showed that computed tomography can facilitate a higher diagnostic accuracy for scaphoid fractures than radiography^15^. The main difference between MDCT and CBCT is the employed detector technology^16^. CBCT has a higher spatial resolution than MDCT^17^, which could in principal allow better visualization of scaphoid fractures. However, CBCT is also more susceptible to motion artifacts^16^, which could hinder diagnostics. In our study, where patients’ wrists were fixed to the CBCT-table, no diagnostic limitations in terms of movement artifacts were identified in the CBCT images. The relatively high number of scanner related artifacts in this study most likely relates to ring artifacts scarcely visible and irrelevant for diagnostics. In our experience, images in the CBCT also frequently show hardening artifacts around the cortical or articular surface, in particular the distal radial joint surface. In our study, however, there was no diagnostic constraint by these artifacts. Our results confirm existing data on the MDCT in that CT is better suited for the diagnosis of scaphoid fractures than radiography^15,18^.

Contrary to our results a recent prospective study employing MRI as a reference standard found no significant difference between the sensitivity of CBCT and radiography in the detection of scaphoid fractures^14^. This may be due to the fact that that study was powered to detect a difference in sensitivity of 15% between radiography and CBCT^14^. However, our data suggest that this difference might not be as high and range around 6%.

The overall good performance of CBCT points to its potential benefit in the diagnostic workup of scaphoid fractures. Nowadays, treatment planning of scaphoid fractures is based on a modified Herbert classification system, meaning that A-fractures can be treated conservatively and B-fractures are treated surgically^19^. In our study, CBCT imaging resulted in 15% treatment-relevant change in fracture classification (A vs. B) compared to radiography, consisting of 7% upgrading and 8% downgrading. In the case of clinically suspected scaphoid fractures and negative radiographs, CBCT can also be considered a modality for further evaluation. Although our data do not cover this clinical scenario, the CBCT, as a low-dose imaging technique, could also be considered a primary diagnostic tool in patients with clinically suspected scaphoid fracture. This has already been discussed for MDCT^20^. Further studies are necessary to evaluate this potential role of the CBCT.

The main limitation of our study is a possible selection bias in patient recruitment due to the retrospective design of the study. Given our inclusion criteria, patients with ambiguous radiographic findings could have been preferentially included, which in turn could have over-emphasized the superiority of CBCT as a problem-solver (by making conventional radiography appear worse than it actually is for the detection of scaphoid fractures). There are two reasons that suggest that this selection bias might be relatively low. Firstly, in our institution all scaphoid fractures diagnosed in the X-ray are actually examined in the CBCT in order to enable an adequate classification of the fractures. Secondly, the prevalence of scaphoid fractures and the diagnostic accuracy for conventional radiography reported in our study agree well with previously-published data^1,2^. However, we cannot definitively quantify the selection bias and its impact on the predictive values cannot be finally estimated. We therefore argue that a prospective study is required to validate the superior diagnostic performance of CBCT.

To conclude, we show that CBCT has a higher diagnostic accuracy for scaphoid fractures than radiography. In the clinical scenario of suspected scaphoid fracture and negative radiographs, CBCT, as a new low-dose technique in trauma imaging of the extremities, may have a substantial benefit for the diagnostic workup.

## Methods

The Ethics Committee of the Albert-Ludwig University of Freiburg approved this retrospective study and approved that informed consent was waived (application number: 175/16). This study is HIPAA compliant and all methods were performed in accordance with the relevant guidelines and regulations in our medical centre.

### Participants

Patients were identified through our electronic health record system. We included all patients who underwent CBCT of the wrist from November 2012 to November 2014 in our institution. We excluded all patients without radiography examinations (scaphoid series) acquired four days before or after the CBCT exam. In our institution all patients with suspected scaphoid fracture are followed with prompt CBCT. We only included patients on whom CBCT was performed because of suspected scaphoid fracture; all other referral reasons for CBCT were excluded. For study inclusion, we required a complete electronic health record. These resulting patient records form a consecutive series.

### Index tests

Digital radiography (scaphoid series) included dorsopalmar, dorsopalmar with ulnar deviation and lateral projections and was performed with 55 kVp and automatic exposure control. CBCT was performed at 90 kVp and 36 mAs (Verity; Planmed, Helsinki, Finland). The patient’s wrist was fixed to the CBCT-table with a tape for immobilization purposes. Axial images were reconstructed with a matrix of 801 × 801, a slice thickness and sparing of 0.2 mm.

The studies were anonymized and randomized. The clinical information was not available to the observers. Two radiologists and two orthopedic surgeons (5–10 years of experience in musculoskeletal radiography and CT) independently interpreted the studies on dedicated workstations. CBCT examinations were read at least 6 weeks after the radiographs, patient order was again randomized and different from the order in which the radiographs were evaluated. Readers evaluated the presence or absence of a scaphoid fracture, the certainty regarding fracture evaluation (Likert scale from 1 = very high to 5 = very low), scaphoid fracture classification (Herbert Classification^19^ from A1 to B5), certainty regarding fracture classification evaluation (Likert scale from 1 = very high to 5 = very low), image quality (Likert scale with 1 = good, 2 = minimal complaint without diagnostic limitation, 3 = bad with diagnostic limitation) and reasons for restricted image quality (wrong rotation, superimposition of foreign material and incomplete depiction/out of field of view, physics-based artifacts, patient-based/motion artifacts, scanner-based artifacts and incomplete depiction^21^, multiple nominations were possible).

### Reference test

Two radiologists (5 & 11 years of experience in musculoskeletal radiography and CT) performed a consensus reading of all imaging studies for fractures and fracture classification in all patients. The imaging studies included radiographs and CBCT for all patients and MRI examinations for 10 patients. The complete electronic health records including the complete clinical course of all patients but not the index test results were available to the assessors.

### Statistical analysis

Pooled sensitivity, specificity, negative predictive value and positive predictive values regarding scaphoid fractures were calculated and compared with McNemar’s test and generalized score statistic. Inter-rater correlation for fracture detection was evaluated with Fleiss’s kappa, bootstrapping (replicates: 1000) was applied to calculate confidence intervals and compare with Student’s t-Test. Correlation of the fracture classification with the reference standard was evaluated with Spearman’s ρ, bootstrapping (replicates: 1000) was applied to calculate confidence intervals and compare with Student’s t-Test. Ratings of image quality, certainty regarding fracture evaluation and certainty regarding fracture classification evaluation were compared with exact Wilcoxon-Mann-Whitney test. Confidence intervals (CI) were stated at 95% level. The Bonferroni Holm method was applied to account for alpha error accumulation. A P-value < 0.05 was considered to denote statistical significance. Statistical analysis was performed with R^22,23^.

### Data Availability

All data generated or analysed during this study are included in this published article.
